# Immunreconstitution and Infectious Complications After Rituximab Treatment in Children and Adolescents: What Do We Know and What Can We Learn from Adults?

**DOI:** 10.3390/cancers7010305

**Published:** 2015-01-29

**Authors:** Jennifer Worch, Olga Makarova, Birgit Burkhardt

**Affiliations:** Pediatric Hematology and Oncology, University Children’s Hospital Münster, Münster, 48149, Germany; E-Mails: jennifer.worch@ukmuenster.de (J.W.); olga.makarova@ukmuenster.de (O.M.)

**Keywords:** rituximab, immunreconstitution, infections, children, adolescents

## Abstract

Rituximab, an anti CD20 monoclonal antibody, is widely used in the treatment of B-cell malignancies in adults and increasingly in pediatric patients. By depleting B-cells, rituximab interferes with humoral immunity. This review provides a comprehensive overview of immune reconstitution and infectious complications after rituximab treatment in children and adolescents. Immune reconstitution starts usually after six months with recovery to normal between nine to twelve months. Extended rituximab treatment results in a prolonged recovery of B-cells without an increase of clinically relevant infections. The kinetic of B-cell recovery is influenced by the concomitant chemotherapy and the underlying disease. Intensive B-NHL treatment such as high-dose chemotherapy followed by rituximab bears a risk for prolonged hypogammaglobulinemia. Overall transient alteration of immune reconstitution and infections after rituximab treatment are acceptable for children and adolescent without significant differences compared to adults. However, age related disparities in the kinetic of immune reconstitution and the definitive role of rituximab in the treatment for children and adolescents with B-cell malignancies need to be evaluated in prospective controlled clinical trials.

## 1. Introduction

Rituximab is a chimeric human-mouse monoclonal antibody that reacts specifically with the CD20 antigen expressed on normal and neoplastic B lymphocytes. CD20 is a membrane embedded protein and a B-cell signature differentiation antigen that appears to regulate the cell cycle and cell differentiation. Different mechanisms of action for rituximab include complement-dependent cytotoxicity (CDC), antibody-dependent cellular cytotoxicity (ADCC) and direct induction of apoptosis [1]. Other mechanisms such as phagocytosis and complement-enhanced ADCC (CR3-ADCC) are also discussed [2,3,4]. Binding of rituximab to CD20 causes rapid depletion of B-cells [5,6]. This is followed by a new ontogeny repopulation of the B cell pool characterized first by the appearance of immature cells (CD38, CD10, CD24), later naïve B cells and finally CD27 memory B cell. Details of characteristics in the reconstituting B cell pool following B cell depletion are still unclear.

Combinations of the above mentioned effector mechanisms might be responsible for the anti-lymphoma action of rituximab [1,7,8]. In the treatment of B-cell malignancies in adults, rituximab is effective and well established. It is also being used for the treatment of autoimmune disorders and .as part of the conditioning regimen in hematopoietic stem cell transplantation [9,10]. The potential risk of infections after rituximab treatment is difficult to quantify because of concomitant use of immunosuppressive or chemotherapeutic agents. Different underlying conditions, different dosing schedules of rituximab and varying definitions of B-cell recovery complicate a valid comparison of several trials. By causing B-cell depletion, rituximab interferes with humoral immunity. Therefore, rituximab might increase the risk of episodes of bacteremia, sepsis, sinopulmonary infections and other opportunistic infections including reactivation of herpes viruses, progression of latent viral infections such as hepatitis B and development of progressive multifocal leukencephalopathy (PML) [11,12,13,14].

Despite increased use of rituximab in the treatment of children and adolescents including pediatric patients with B-cell malignancies, data on the impact of rituximab on the immune system and infectious complications are limited. If available, observations on immunological effects especially immunoglobulin levels and vaccination titer in children and adolescents are often described in case reports or small case series. Details of the reconstitution of the B cell pool and the question whether rituximab treatment results in more severe immunological late effects when administered to young patients with a more immature immune system are addressed in the current review.

### 1.1. Immunreconstitution after Rituximab Treatment in Children and Adolescents

Most publications summarize the kinetic of B-cell recovery and immunoglobulin level under the term “immunreconstitution”. However, there are no available generally accepted definitions, which hampers the comparison of the available data. Concerning children with B-cell malignancies, data on the immunreconstitution are limited. Due to small numbers of patients treated with the antibody in systematic clinical trials, single institution case series have been reported for these patients, but comprehensive data analyses are overall lacking. Many of these reports include children who received rituximab for non-malignant disorders. The drug has been frequently used for children with autoimmune diseases and after organ transplantation. The kinetic of B-cell depletion and recovery was nicely reported in two papers on 35 children and adolescents who were treated for hematologic autoimmune cytopenia, nephrotic syndrome and acute rejection after renal transplantation and who showed a depletion of B-cells with recovery about 6–12 months after treatment with rituximab 375 mg/m^2^ weekly with 1–4 doses [15,16]. Concerning the impact of the rituximab dosing schedule on the duration of this transient B-cell depletion there was no difference between single and repeated doses of rituximab. Concerning the question, whether the addition of rituximab at all prolongs B-cell recovery, another randomized prospective trial of rituximab for acute rejection in 20 pediatric renal transplantation patients reported no difference between the standard-of care rejection therapy or the standard-of care therapy combined with four weekly doses of rituximab. Repopulation of B-cells after complete depletion was seen at a mean time of 11.8 months after the end rituximab therapy. Interestingly, there was a strong correlation with the recipient age: B-cells recovered faster in children less than ten years of age compared to children older than 10 years (5 months* versus* 14 months) [17].

As B-cells are responsible for immunoglobulin production it is expected that B-cell depletion might alter immunoglobulin levels. Interestingly the data available on immunoglobulin levels after rituximab treatment in children and adolescents with non-malignant disease are inhomogeneous. While some reported decreased immunoglobulin levels that remain within the normal range, others reported mild hypogammaglobulinemia with immunoglobulin concentrations that fell below the age-adjusted values as summarized in Table 1. One of the largest and most detailed analyses of pediatric patients with rituximab treatment summarized 91 pediatric patients with immune thrombocytopenia. Among those, 108 adverse events were documented of which 84% were mild to moderate with only one patient who developed common variable immunodeficiency with prolonged hypogammaglobulinemia [18]. One publication reported a significant decrease of IgM levels but IgG levels without relevant hypogammaglobulinemia [19]. The heterogeneous picture of immune reconstitution may broadly reflect differences in the underlying disease, varying rituximab schedules and differences in pre-treatment or concomitant therapy. However, regarding children and adolescents with non-malignant diseases, no major immunological or infectious complications after rituximab treatment have been published.

In contrast to children with non-malignant disease, the status of the immune system prior treatment and the kinetic of recovery was reported to be more heavily altered in children with underlying malignant diseases [20]. Regarding children with malignant diseases receiving rituximab treatment, the Non-Hodgkin-Lymphoma Berlin-Frankfurt-Münster (NHL-BFM) group reported serum immunoglobulin levels in 344 pediatric B-NHL patients before and after chemotherapy with and without rituximab [21]. Interestingly approximately one third of patients already presented with hypogammaglobulinemia at initial diagnosis prior to the start of treatment, which fits into the observation of Mellgren and colleagues reporting disease induced changes of immune regulation in newly diagnosed NHL patients [20]. For the NHL-BFM patients, hypogammaglobulinemia about 12 months after start of therapy was reported for about 50% of the patients without significant differences between patients receiving chemotherapy alone or chemotherapy combined with one dose of rituximab. Among those patients with Ig levels below normal, isolated decreases of IgM, IgG and IgA were reported in 22%, 16% and 12% of patients, respectively. In the remaining 50% of patients, more than one subclass was below normal limits. Patients with hypogammaglobulinemia presented asymptomatic in 75% and with frequent but mostly mild infections in 19% (no data in 6%). Patients in the high risk group R3/R4 receiving more intense treatment presented more frequently with hypogammaglobulinemia compared to patients in the low risk group R2. But in both subgroups the Ig levels were not significantly different between patients who had chemotherapy only or chemotherapy plus one dose of rituximab [21]. Also Abrahamsson and colleagues described hypogammaglobulinemia in pediatric lymphoma patients after chemotherapeutic treatment without rituximab [22]. In conclusion, the observation of altered immunoglobulin levels in pediatric patients might be influenced by host factors as for example the underlying disease and the cytokine release status on the one hand, and on the chemotherapeutic agents and rituximab administration schedule on the other hand. To further analyze this observation we searched for publications reporting the kinetic of immune reconstitution in children and adolescents who received even more intense treatment like myeloablative high dose chemotherapy and stem cell transplantation. It was reported for adult patients that intensive treatment with rituximab as part of high dose chemotherapy and stem cell transplantation (SCT) or maintenance after SCT bears the risk for prolonged hypogammaglobulinemia [23,24,25,26,27]. Regarding pediatric reports Kano and colleagues reported the course of a 10-year old girl who developed persistent hypogammaglubilinemia and complications with massive lymphadenopathy following eight rituximab containing chemotherapy courses as salvage therapy for relapsed B-NHL. Interestingly, the patient’s B-cell phenotype with decreased memory cells and increased transitional B-cells was similar to that of common variable immune deficiency syndrome [28]. Another indication of rituximab treatment in the context of SCT is the Epstein-Barr virus induced post-transplant lymphoproliferative disease (PTLD) which can be effectively treated by rituximab [29]. A case report described long lasting hypogammaglobulinemia with low levels of CD20+ lymphocytes for more than two years after rituximab infusion for high-level EBV in a 9-year old boy [30]. Masjosthusmann and colleagues compared six consecutive pediatric allogenic SCT patients treated with rituximab with a matched control group of non-rituximab treated patients. Substitution of intravenous immunoglobulin and the mean time to recovery was prolonged in the rituximab group compared to the control group. Five of six patients with rituximab recovered within one year after treatment but one patient suffered isolated B-cell deficiency with persistent hypogammaglobulinemia lasting for more than three years post transplantation [31]. Kuehnle and colleagues report three children with rituximab treatment for post SCT PTLD. Two patients had normal B-cell numbers and levels of immunoglobulins 7 and 9 months after administration of rituximab therapy whereas the third patient continued to have profound B-cell deficiency and hypogammaglobulinemie eight months after rituximab. None of the patients had an increase in opportunistic infections [32].

In conclusion the available data on the kinetic of immune reconstitution after rituximab treatment in children and adolescents are limited, but hint at an essential impact of the underlying disease, the concomitant treatment and the maturation status of the immune system. In most cases the immunoglobulin levels recover within one year without serious toxicities, but single cases of prolonged hypogammaglobulinemia have been described.

### 1.2. Immune Response and Vaccination Titres after Rituximab Treatment

Since rituximab causes B-cell depletion, in can be hypothesized that rituximab treatment alters the response to vaccination [33]. Some studies described lower response of the humoral immune system to vaccines after rituximab treatment first in nonhuman primates and later in adult patients with autoimmune disease [34,35,36]. A randomized controlled trial examined immunization response in adult patients with rheumatoid arthritis treated either with rituximab plus methotrexate (MTX) or MTX alone. The response to tetanus vaccine was tested to evaluate the integrity of the T-cell dependent anamnestic humoral response and the pneumococcal polysaccharide vaccine was used to evaluate the T-cell independent B-cell response. The recall responses to tetanus toxoid were similar in both patient groups; those with and without rituximab treatment. Rituximab treated patients showed a decreased response to pneumococcal vaccine, but still many patients were able to mount responses [37]. Regarding data obtained in patients with malignant diseases, humoral response to influenza vaccine was impaired in lymphoma patients treated with rituximab containing regimens compared with age-matched healthy subjects [38,39]. Reduction of CD27 positive memory B cells and low immunoglobulin levels among these rituximab treated patients correlated with a less effective response to influenza vaccination. Therefore, perturbation and reduction of B-cells and immunoglobulin synthesis might be a mechanism contributing to the impaired humoral immune response [38]. Interestingly in lymphoma patients without rituximab treatment, humoral response to influenza vaccination was reported to be weaker compared with healthy controls irrespective of treatment indicating the impact of the underlying disease on the immune system [40,41,42,43,44].

Concerning the question which factors except the underlying diseases impact on the humoral response to vaccination after rituximab treatment, it was shown that vaccination response was more likely in systemic lupus erythematosus (SLE) patients with earlier reconstitution of B-cells [45]. From data reported for patients who received rituximab for autoimmune diseases or lymphoma, it was speculated that the observed decrease in the responses to recall antigens might be attributed to a decrease in the amount of memory B cells [46]. So the ability to mount a response to antigens seems to increase with the passing of time after rituximab treatment [37]. This is in line with the observation of a modestly restored humoral response following influenza vaccination 6–10 months after rituximab administration in adult patients with rheumatoid arthritis [47]. Also in lymphoma patients increasing B-cell counts were significantly associated with a more effective response to influenza vaccines [38]. So, rituximab induced depletion of C19/CD20 positive B-cells might at least for a certain period hamper the generation of antibodies after exposition to antigens. However, it was reported that tetanus toxoid antibody titers were detectable throughout the follow up of one year despite undetectable B-cells for three months after rituximab treatment for pediatric autoimmune cytopenias [15]. Furthermore, Hassan and colleagues reported a phase II study including five adult patients treated with an immunotoxin targeting the Lewis Y tumor antigen in combination with rituximab. The aim of adding rituximab was to prohibit the generation of neutralizing antibodies against the immunotoxine. Interestingly, all patients developed neutralizing antibodies despite absent circulating antibody-producing B cells [48]. Therefore, additional factors like the potency of the antigens have impact on the immune response after rituximab treatment and might be relevant for humoral response.

These heterogeneous pictures of the above cited papers underline the necessity of prospective clinical trials with repeated antibody determination before and after treatment like performed for children with acute lymphoblastic leukemia to systematically evaluate the functional read out of B-cells measured as response to vaccinations in children and adolescents receiving rituximab added to chemotherapy for lymphoma [49,50]. We have re-edited your manuscript according to our layout style.

**Table 1:** Immunreconstitution and immunoglobulin levels after rituximab therapy in children and adolescents with non-malignant diseases.

Diagnosis and Numbers	B-cell Reconstitution	Ig Level, Vaccination	Reference
chronic or refractory hematologic autoimmune cytopenias, rituximab 4 doses 375 mg/m^2^, non-responder after 3 doses: escalation to 3 × 750 mg/m², total 29 pts with 9 pts with dose escalation	B-cell recovery was noticed after 6 months. B cells were normalized after 1 year. Recovery was similar in pts that received 4 or 6 doses of rituxmab	IgM, IgA, IgG decreased but remained near normal range	[15]
autoimmune haemolytic anaemia, rituxmab 4 weekly doses 375 mg/m², 6 pts. 2 pts received 8 additional infusions	B cells did not reappear in blood 5–9 months after last infusion. Normal counts were then reached within the following months “A reduction to one or two injections, which would limit the duration of B-cell deficiency would be worth assessing”	Ig concentrations in serum fell below normal values for age. 5 pts were substituted 9–10 months after last ritux mab infusion	[16]
autoimmune hemolytic anemia (n = 9) and Evans syndrome (n = 6), ritux 375 mg/m² weekly with 2 doses (n = 3), 3 doses (n = 10), 4 doses (n = 2), 15 pts	B-cell undetectable after treatment in all pts. Normal B-cell count after 6 mo in 10/15 (67%) of pts		[51]
nephrotic syndrome, single dose ritux 375 mg/m², 12 pts	Median time to B-cell recovery 119 days	Serum IgM levels gradually decreased	[52]
nephrotic syndrome, rituxmab 375 mg/m² weekly in 4 doses (n = 15), 3 doses (n = 2), 2 doses (n = 4) or 1 dose (n = 1), re-treatment single dose (n = 19), 22 pts	Duration of complete B-cell depletion 3 to 15 mths (mean 8 mths). Similar duration of B-cell depletion in pts with 1–2 doses and in pts with 3–4 doses of rituxmab		[53]
acute rejection after renal transplantation, 4 doses 375 mg/m² weekly, 20 randomized pts	Correlation of age and B-cell recovery: children < 10years of age repopulated significantly faster than children > 10years of age (5 *vs.* 14 months)	IgM levels trended lower in rituximab group compared to control. Correlation between lowering of serum IgM, young age and B-cell repopulation > 10 months	[17]
severe chronic ITP, rituxmab 375 mg/m² weekly for 4 doses, 36 pts	B cell depletion in all pts, remaining unchanged at 2% between week 6 and week 12	No significant HG: mean IgG falling only 0.7%/week but significant decrease of mean IgM levels. “it would appear that IVIG replacement therapy is unnecessary.”	[19]
Chronic ITP, rituxmab 375 mg/m² weekly for 4 doses, 24 pts		decreased IgG in 4 pts, decreased IgM in 5 pts	[54]
Chronic ITP, rituxmab 375 mg/m² weekly for 4 doses, single pt	low CD19-positive cells (<400 × 10^9^/L)	after begin of rituximab, IgG, IgM and IgA level were decreased for 3 years, with only increased IgG thereafter	[55]
SLE, rituxmab 750 mg/m² 1 dose (19 pts had 2 -6 doses), 63 pts		After a mean of 2.5 months, IgG, IgA and IgM levels were reduced, but only 2% with Ig replacement	[56]
autoimmune and inflammatory CNS disease, 144 pts, rituximab 375 mg/m^2^ 1–10 doses, the most common regimen weekly for 4 weeks (n = 57)	B-cell depletion in 119/24 (96%), >12mo in 12/124 (10%)	Hypogammaglobulinemia in 27/124 (22%)	[57]

Abbreviations: ITP: Idiopathic thrombocytopenic purpura; SLE: Systemic lupus erythematosus; CNS: Central nervous system; PTS: Patients; L: Liter; N: Number; IVIG: Intravenous immunoglobulin substitution.

### 1.3. Immunereconstitution in Adult B-NHL Patients Treated with Rituximab and Chemotherapy: Lessons for the Pediatricians

**Figure 1:**
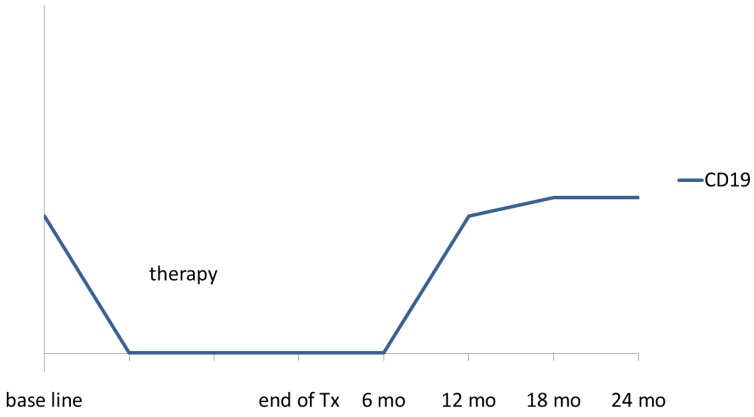
Schematic presentation of CD19 lymphocytes count in peripheral blood in NHL patients treated with rituximab.

**Figure 2:**
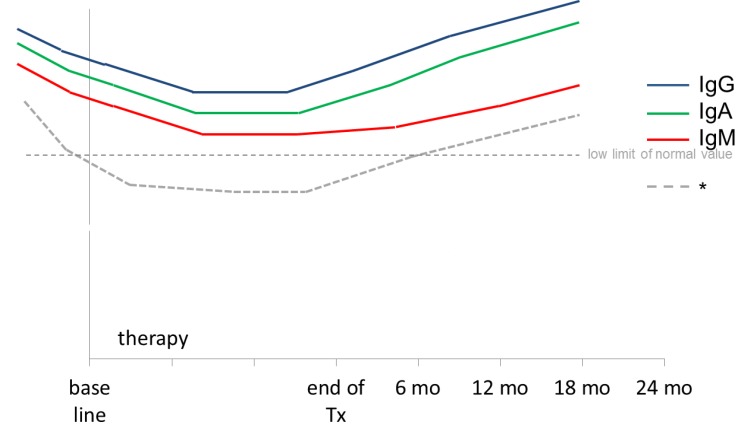
Schematic presentation of immunoglobulin levels in NHL patients treated with rituximab. Hypogammaglobulinemia observed in 40%–50% of patients after rituximab therapy [59,68].

The effect of rituximab on the immune reconstitution might vary for children and adolescents on the one hand and adults on the other hands, due to differences in the maturation status of the immune system, in the biology of the underlying disease and in the chemotherapy regimens including varying dosing schedules of rituximab. However, the experiences of adult oncologists on rituximab might serve as an orientation for pediatric oncologists. Table 2 provides an overview of literature on the immune reconstitution and immunoglobulin levels of adult NHL patients treated with different doses of rituximab as single agent or combined with chemotherapy. Concerning B-cell counts, rituximab treatment induced a rapid B-cell depletion of in the peripheral blood with recovery about 6–12 months after rituximab treatment without differences in the duration of transient B-cell depletion according to single or repeated dosing of rituximab. In adults serum immunoglobulin levels stayed mostly within the normal limit or in the level of mild hypogammaglobulinemia [58,59,60] and recovered to normal within one year [58,59,60,61]. Figure 1 and Figure 2 present schematically the kinetic of CD19 lymphocyte counts and peripheral blood immunoglobulin levels in NHL patients after treatment with rituximab.

In adult patients, additional doses of rituximab after the rituximab standard of four doses resulted in a slightly prolonged median recovery of B-cells to baseline values, but overall B-cells increased between 12 and 18 months after therapy [61,62,63]. Importantly, longer immunosuppression was not associated with an increase of clinically relevant infections [61].

As mentioned earlier for children and adolescents, clinically most relevant seems the delay of immune reconstitution in patients who receive rituximab in the context of a high-dose chemotherapy and SCT or even worse, as treatment of post-transplant EBV induced PTLD. After pretreatment with rituximab before SCT in adults, B-cell counts normalised between 18 and 24 months [27,64]. Incidence of lymphopenia was not different comparing patients with similar chemotherapy with or without adjuvant rituximab [24]. However, cases of prolonged hypogammaglobulinemia after SCT followed by rituximab treatment are described. Nine patients with high risk lymphoma, who received maintenance rituximab after SCT demonstrated low immunoglobulin levels below 50% of normal immunoglobulin levels for IgM up to two years after transplant [65,66]. Profound panhypogammaglobulinemia with very low levels of IgA, IgG and IgM for 7 years after combined treatment for chemotherapy and rituximab for follicular lymphoma was reported in a 31-year old women. In the setting of high-dose chemotherapy and SCT for aplastic anemia followed by rituximab treatment for PTLD Imashuku described an adult case of hypogammaglobulinemia for more than 2.5 years. This hypogammaglobulinemia persisted and required continuous replacement therapy with intravenous Ig [67]. These single cases emphasize the risk of prolonged impaired immunoglobulin production which might be at least in part be attributed to rituximab treatment in the setting of a recovering bone marrow and immune system after high-dose chemotherapy and bone marrow transplant.

## 2. Infectious Complications after Rituximab Treatment

Given the known effect of B-cell depletion, the alteration of response to vaccinations and -at least in children- the decrease of immunoglobulin levels after rituximab treatment, the fear of causing additional infectious complications contributes to the more reluctant use of rituximab in children and adolescents compared to adults. B-cell counts, immunoglobulin levels and vaccination titres are laboratory values of minor clinical relevance, but what is the clinically relevant risk of adding rituximab to standard treatment of children and adolescents?

**Table 2:** Immunreconstitution in adult NHL patients after rituximab therapy.

Diagnosis and Numbers	B-cell Reconstitution	Ig Level, Comments	Reference
relapsed FL, 4 doses ritux 375 mg/m² (n = 7), de novo FL 4 doses (n = 4)	B-cell reconstitution (>5 cells/µL) 6–9 months, recovery to pre-treatment levels by 12 months	delayed recovery of CD27+ memory B-cell pool; emerging B cells after rituxmab are functionally immature	[69]
FL and MCL, 4 weekly doses of rituximab mono 375 mg/m² (arm A)	1 year after start median B-cell level 81% of baseline (n = 22)	IgG and IgM remained stable. IgM 100% recovered after 1 year (n = 30)	[61]
4 weekly doses and 4 more doses at 2-month intervals (arm B)	1 year after start median B-cell level 50% of baseline, 6 months longer to recover to baseline (n = 35)	IgG remained stable. IgM decreased to 73% of baseline after 1 year (n = 50) (p 0.007)	
relapsed FL, 4 weekly doses 375 mg/m² of rituximab mono (n = 166)	recovery of B cells started between 6 and 9 months; recovery to normal between 9 and 12 months	Mean serum IgA and IgG levels remained within normal. IgM recovered to normal at 8 mths	[58]
B-NHL (n = 66), 6× CHOP + rituxmab 375 mg/m² prior each cycle + 2 doses within 1 month (total 8 doses rituximab)	After 1 cycle R-CHOP CD19+ and CD20+ completely eliminated. One year after therapy B-cell levels same as at diagnosis and were almost double the level at diagnosis 2 years after therapy	After 6 cycles: IgG 68%, IgA 63%, IgM 56%. All Ig recovered gradually until 2 years after therapy IgG 94%, IgA 90%, IgM 76%.	[63]
FL or low grade Lymphoma, 8 doses rituximab 375 mg/m^2^ weekly, 37 pts	B-cell with counts returning to the lower limit of normal recovery 6–9 months after rituximab	Mean serum IgG, IgA and IgM levels within normal range. Decrease > 50% from baseline in IgG, IgM or IgA was observed in two, one and four pts, respectively. Within 1 year, levels recovered in all but one pt (low IgG)	[60]
review of different diagnoses	reconstitution of B lymphocytes usually takes several months; return to pre-treatment value after about one year	Reduction of serum Ig levels; unclear whether clinically significant	[70]
B-cell lymphoma (n = 211), median of 7× rituximab (monotherapy or combined with chemotherapy)		39% of patient with initially normal IgG levels presented with hypogammaglobulinemia (mild for 77% of pt).	[59]
Diffuse large B-cell lymphoma (n = 122) treated with CHOP (n = 24) or rituximab-CHOP (n = 98); 16 pts were excluded due to abnormal Ig levels before treatment; 6 cycles of treatment		No obvious changes of IgG, IgA and IgM in the CHOP group. Decreased levels of IgG, IgA and IgM by 20% of baseline in 85%, 85% and 88% respectively. IgG, IgA and IgM < low limit of normal value were observed in 48%, 49% and 52% of pts, respectively.	[68]
B-cell NHL (n = 66). Standart treatment included R-CHOP, CHOP and CVP. Exact number of rituximab infusions is not indicated		Levels for IgA, Igg and IgM were decreased after treatment compared to pretreament levels. No occurrence of higher infection rates	[71]

Abbreviations: NHL: Non Hodgkin lymphoma; FL: Follicular lymphoma; MCL: Mantle cell lymphoma; R-CHOP: Rituximab, Cyclophosphamide, Hydroxydaunorubicin, Oncovin (Vincristine), Prednisone; CHOP: Cyclophosphamide, Hydroxydaunorubicin, Oncovin (Vincristine), Prednisone; CVP: Cyclophosphamide, Vincristine, Prednisolone; PTS: Patients.

### 2.1. Infectious Complications after Rituximab Treatment in Children and Adolescents

The potential risk of infection after rituximab treatment is difficult to quantify because of concomitant use of immunosuppressive or chemotherapeutic agents and different underlying conditions. Reports evaluating rates of infectious complications after use of rituximab in children and adolescents are rare. This might be related to the small number of patients treated within clinical trials. A recent retrospective cohort study identified 2875 pediatric patients with 4639 rituximab admissions at 42 children’s hospitals across the United States [11]. Administrative data from the Pediatric Health Information System were analysed to estimate the frequency of infectious events within a 1-year period after rituximab exposure. According to the underlying disease all evaluated patients (n = 2875) were categorized into transplant (n = 1163), malignancy (n = 479), primary immunodeficiency (n = 105), autoimmune disease (n = 764) and others (n =364). Of the patients in the malignancy category, 1–4 admissions for rituximab during the 1-year follow-up period were noted for 35%, 17%, 19%, and 20% of patients, respectively. At least one infectious complication within one year after rituximab exposure including bacterial, viral, and invasive fungal infections was distributed similarly across the transplant and malignant category. Proportions of patients with at least one bacterial, viral or invasive fungal infection within one year after the rituximab exposure were similarly distributed across the transplant and malignant category. Patients in the primary immunodeficiency category had highest rates of infections (sepsis, cytomegalovirus, adenovirus infection) and those in the autoimmune category had the lowest. In total, the rates of opportunistic infections appear to be low with three cases with a discharge diagnosis code for *P. joroveci* pneumonia, one patient with hepatitis B and one with progressive multifocal leukoencephalopathy.

In children and adolescents who received rituximab for non-malignant disorders like hematologic autoimmune cytopenia, nephrotic syndrome and acute rejection after renal transplantation early or delayed infectious complications were rarely reported. In a prospective study of 36 children with severe chronic thrombocytopenic purpura (ITP), rituximab treatment was well tolerated. One patient developed primary varicella after the first rituximab infusion and recovered completely after adequate therapy. No other serious infections occurred [19]. Toxicity and serious adverse events including infections and published details of rituximab treatment in pediatric patients with other indications than B-cell lymphoma are summarized in Table 3 below.

Observations of infectious complications in children with malignant diseases and rituximab treatment are mostly reported in case reports or small case series. Griffin and colleagues reported results of rituximab combined to standard salvage chemotherapy including ifosfamide, carboplatin and etoposide in 20 pediatric patients (5–20 years of age) with relapsed and refractory B-cell Non-Hodgkin lymphoma/leukemia. As expected at that stage of treatment and treatment intensity, infections were common but manageable and no patient died of infection. Whether rituximab added an additional risk to the intensive chemotherapy remained open [72].

The feasibility of adding rituximab to a modified NHL-BFM90 protocol in pediatric patients with advanced-stage mature B-NHL was reported by Samochatova and colleagues [73,74] Differences to the original NHL-BFM90 protocol were the dose of methotrexate which was reduced from 5000 to 1000 mg/m² in the first two courses and the addition of 375 mg/m² rituximab to each of the first four courses of therapy Eighty-three newly diagnosed Burkitt or DLBCL lymphoma patients (median age 8.8 years) with stage III to IV disease were included. Four patients died during the first month after initial chemotherapy due to tumor lysis syndrome and infection during myelosuppression. None of the patients died thereafter. It is not stated whether infection is possibly related to rituximab [74]. As mentioned earlier in this review the NHL-BFM group investigated rituximab in a phase II trial with an upfront window of one dose rituximab monotherapy five days before the start of standard chemotherapy in newly diagnosed pediatric B-cell lymphoma/leukemia patients [75]. One hundred and thirty six (136) patients below 19 years of age were enrolled and treated on day one with rituximab at 375 mg/m^2^. There were no hepatitis B reactivations, no progressive multifocal leukencephalopathy, and no therapy related deaths. Serious adverse events included—except for intestinal perforation with subsequent peritonitis—no case of infection. All patients recovered without late effects. For additional 64 patients the dose of rituximab was increased to 700 mg/m² as a single dose five days before the start of standard chemotherapy [76]. The rate of infection of the increased rituximab dose did not differ from the lower dose of 3% infections. No toxic death was reported. The COG evaluated the addition of rituximab to frontline FAB-based chemotherapy in children, adolescents and young adults (<30 years) with St. Jude Stages III/IV mature B-cell B-NHL. Patients received a total of six rituximab doses except for a pilot-patient subgroup with a total of four doses of rituximab. In 51 patients with intermediate risk (FAB group B), no toxic deaths were reported. No serious adverse event was probably or definitely attributed to rituximab. One episode of grade III infectious colitis occurred after the second induction cycle and was attributed as possibly related to rituximab administration [77]. For high risk (FAB group C), Goldman and colleagues reported in 40 pediatric patients only one grade 3 infusion reaction definitely attributed to rituximab [78]. The rate of toxic death at 5% was nearly identical to the rates reported in the same population of patients treated with FAB96 C1 without rituximab [79,80]. One patient died of pre-existing pulmonary aspergillosis. The second patient death was secondary to mucositis/typhylitis and sepsis. Although rituximab may have contributed to the additional mucosal breakdown and resultant sepsis, more relevant seems that the patient did not receive the required leucovorin rescue following high dose methotrexate.

A review from 2008 by Attias and Weitzman evaluated all published data on rituximab therapy for Burkitt lymphoma/B leukaemia (B-AL) and pediatric patients with relapsed/refractory large B-cell lymphoma [81]. Nineteen cases of children with high-grade B-lineage disease who received rituximab alone or in combination with chemotherapy as salvage therapy were identified. Infectious complications were not reported. Further data on childhood B-NHL are mainly case reports.

### 2.2. Relevant Infectious Complications Reported in Adult Patients after Rituximab Treatment

Given the much higher frequency of rituximab administrations in adult patients, much more reports on infectious complications after rituximab or rituximab in combination with other drugs, are available. Selected manuscripts are summarized in the Table 4 and Table 5. In summary, the addition of rituximab to standard B-NHL treatment in adults seems not be associated with a significant increase of infections [82,83]. In a meta-analysis, the relative risk for fever or leukocytopenia was significantly higher in patients treated with rituximab combined to chemotherapy, but there was no difference between treatment groups with respect to the risk of infection [82]. Table 4 summarizes infectious complications of randomized controlled trials comparing chemotherapy alone* versus* chemotherapy combined with rituximab for NHL treatment.

**Table 3:** Selection of reports on the use of rituximab in children for other indications than B-cell lymphoma.

Indication	No. Pts	Age Years	Dosing	Toxicity	Serious Adverse Events	Reference
nephrotic syndrome	12	<20	1 × 375	mild reactions (n = 5) 42%; fever and hypotension (n = 1), tachycardia (n = 1), hypertension (n = 1), facial flushing(n = 1), mild respiratory distress (n = 1)	no SAE during the patients clinical courses	[52]
nephrotic syndrome	22		375, 4× (n = 15), 3× (n = 2), 2× (n = 4), 1× (n = 1), re-treatment 1× (n = 19)	dizziness, polypnea with dyspnea and tachycardia (n = 2), neutropenia (n = 19), peripheral vein thrombosis (n = 1), transient hepatic cytolysis (n = 1), transient thrombocytopenia (n = 1), gastroenteritis (n = 1), fever (n = 1)	no major side effects were observed	[53]
acute rejection after renal transplant, randomized	20	2–23	4 × 375	mild hypotension and shortness of breath at the first dose only (n = 2)	no SAE	[17]
severe chronic ITP (n = 30) or Evans Syndrom (n = 6)	36		375, 4× (n = 33), <4× (n = 3)	not all 4 doses (n = 3) due to serum sickness (n = 2), infusion related hypotension (n = 1)		[19]
autoimm. hemolytic anemia (n = 9), Evans syndrom (n = 6)	15	0.3–14	375, 2× (n = 3), 3× (n = 10), 4× (n = 2)	infusion-related side effects: fever (n = 2) upper airway edema (n = 1)	primary varicella zoster virus infection two months after rituximab	[51]
Chronic or refractory hematologic autoimm. cytopenia	29	<21	4 × 375, non-responder after 3×, escalation to 3 × 750 (n = 9)	Mild infusion reactions including respiratory symptoms, fever, myalgia. No delayed infusion reactions, no early or delayed infectious complications	one patient (3%) did not tolerate the drug	[15]
autoimm. haemolytic anaemia	6	0.6–2.9	375, 4× (n = 4), 12× (n = 12)	*E. coli* pyelonephriitis (n = 1), febrile bronchitis (n = 1)	no infusion-related side effects, low incidence of infections	[16]
Diamond-Blackfan anemia	1	8	375, 2× weekly		no immediate serious side effect was observed	[84]
autoimmune and inflammatory CNS disease	144	<18	375 mg/m^2^, 1–10 doses	Infectious any 11 (7.6%): 4^0^: 2 (1.4%) CMV retinitis; shock and hypoxic brain injury, 3^0^: 7 (5%): pneumonia (n = 2), empyema, bronchiectasis, salmonella enteritis, C. difficile enteritis, mastoiditis (all n = 1)	Infectious, 5^0^: 2 (1.4), CMV colitis (complicated by fatal bowel perforation); staphylococcus toxic shock syndrome	[57]

ITP: Idiopathic thrombocytopenic purpura; CMV: Cytomegalie virus; SAE: serious adverse event; PTS: Patients.

**Table 4:** Infectious complications in randomized controlled trials comparing chemotherapy alone* versus* chemotherapy combined with rituximab for NHL treatment.

Infection and Complication	Evidence and Diagnosis	Treatment Including Rituximab	Comments and/or Results	Reference
lympho-cytopenia without increased rates of infections	prospective randomized study in relapsed FL and MCL (n = 147)	fludarabine, cyclophosphamide, mitoxantrone with or without rituximab	lymphocytopenia grade III/IV more frequent with R-FCM (51%) compared to FCM (39%; p 0.006) without clinical relevance,* i.e.*, no increased risk of infectious complications; WHO grade III/IV infections of 1.5% were not different between the two arms	[85]
overall infections and neutropenia including long term follow-up	randomized trial untreated DLBCL (n = 399)	CHOP (n = 197)* versus* R-CHOP (n = 202)	similar incidence of 65% for infectious events for all grades, grade III/IV infections were 12% for R-CHOP* versus* 20% for CHOP; neutropenia was not associated with an increase in episodes of infection, number of patients with infections in 5 year follow-up with trend of increase (12 R-CHOP* versus* 6 CHOP)	[86]
overall rates of infections	DLBCL randomized trial (n = 824)	CHOP (n = 411)* versus* R-CHOP (n = 413)	Similar rates of infections for R-CHOP (30%)* versus* CHOP (31%)	[87]
overall rates of infections	DLBCL randomized trial	CHOP (n = 314) *vs.* R-CHOP (n = 318)	Similar rates of grade III/IV infections for R-CHOP* versus* CHOP (17% *vs.* 16%) and neutropenia (78% *vs.* 78%)	[88]
overall rates of infections including fever of unknown origin and hemato-logical toxicities	FL, randomized trial (n = 428)	CHOP (n = 205) *vs.* R-CHOP (n = 223)	Similar rates of grade III/IV infections for R-CHOP *vs.* CHOP (5% *vs.* 7%) despite higher frequency of granulocytopenia grade III/IV with R-CHOP *vs.* CHOP (63% *vs.* 53%)similar numbers of infection-related deaths with both treatment arms	[89]
WHO grade infections	MCL, randomized trial (n = 122)	CHOP (n = 60) *vs.* R-CHOP (n = 62)	Similar grade III/IV infectious complications comparing R-CHOP with CHOP (5% *vs.* 6%)	[90]
hematological and non-hematological toxicity	MCL; phase III randomized study (n = 156)	fludarabine and cyclophosphamide with (n = 78) or without rituximab (n = 78)	toxicity data (n = 139): non-hemotological toxicity similar; more hematological events III/IV with 58% (FCR) leukopenia compared to 41% (FC) leukopenia (p 0.024); this did not translate into increased rates of febrile episodes or infections	[91]

Abbreviations: DLBCL: Diffuse large B-cell lymphoma; FL: Follicular lymphoma; MCL: Mantle cell lymphoma; R-CHOP: Rituximab, Cyclophosphamide, Hydroxydaunorubicin, Oncovin (Vincristine), Prednisone; FCR: Fludarabine, cyclophosphamide, rituximab; FC: Cyclophosphamide, Vincristine, Prednisolone; FCM: Fludarabine, Cyclophosphamide, Mitoxantrone; R-FCM: Rituximab, Fludarabine, Cyclophosphamide, Mitoxantrone.

**Table 5:** Reports on unusual viral infections after treatment with rituximab in adult patients with NHL.

Infection and Complication	Evidence and Diagnosis	Treatment Including Rituximab	Comments and/or Results	Reference
PML	MCL (n = 2) splenic MZL (n = 1)	Rituximab combine d with hyperCVAD and DHAP (n = 1), CVP (n = 1) or CHOP (n = 1)	3 case reports of PML in HIV negative patients	[92]
PML	NHL (n = 976)	rituximab (n = 517), no rituximab (n = 459)	Retrospective cohort study in HIV negative patients	[93]
PML	FL (n = 1), ALL (n = 1), DLBCL (n = 1)	Case 1: CHOP, R-DHAP and ASCT, Case 2: Rituximab, R-P-VABEC, CIP, Case 3: Hyper-CVAD + imatinib, HSCT rituximab	3 case reports of PML in HIV negative patients	[94]
PML	B-cell lympho-proliferative disorders (n = 52); autoimmune disorders (n = 5)	rituximab combined with HSCT (n = 7), purine analogs (n = 26), alkylating agents (n = 39)	clinical characteristics of 52 HIV negative patients with PML, median time from last rituximab dose to PML diagnosis was 5.5 months, overall incidence of fatality 90%	[13]
PML and CMV	mediastinal thymic B-NHL, DLBCL, MZ	HSCT and rituximab	PML (n = 2); CMV retinitis (n = 1); CMV pneumonitis (n = 1)	[95]
PML	DLBCL	R-CHOP	Case report of PML	[96]
PML	electronic medical records from the Veteran’s Administration, 2003–2011, 57,041 non-HIV NHL pts	rituximab, cyclophosphamide, hydroxydaunorubicin vincrisitne	PML: 14/57,041 (0.025%): 7/8895 (7.8 per 10,000) NHL patients who received rituximab 7/48,146 (1.5 per 10,000) NHL patients who did not receive rituximab	[97]

Abbreviations: NHL: Non Hodgkin lymphoma; DLBCL: Diffuse large B-cell lymphoma; FL: Follicular lymphoma; MCL: Mantle cell lymphoma, MZ: Marginal zone lymphoma, ALL: Acute lymphoblastic leukemia, PML: Progressive multifocal leukencephalopathy, CMV: Cytomegalie virus, HIV: Human immunodeficiency virus; CHOP: Cyclophosphamide, Hydroxydaunorubicin, Oncovin (Vincristine), Prednisone, R-CHOP: Rituximab, Cyclophosphamide, Hydroxydaunorubicin, Oncovin (Vincristine), Prednisone, R-DHAP: Rituximab, Dexamethasone, High-dose Ara-C-Cytarabine, Planitol (Cisplatin); R-P-VABEC: Rituximab, Prednisone, Vincristine, Doxorubicin, Bleomycin, Etoposide, Cyclophosphamide, Hyper CVAD: Hyperfractionated Cyclophosphamid, Vincristine, Adriamycin, Dexamethasone, CIP: Cisplatin, Idarubicin, Prednisone, HSCT: Hematopoietic stem cell transplantation, ASCT: Autologous stem cell transplantation.

Critical infectious complications seem to be more common in patients with severe immunodeficiency caused by HIV infection, immunosuppressive agents like fludarabine, heavy pre-treatment or advanced immune compromise [98,99]. Reported infectious complications in these patients include hepatitis B reactivation, pneumocystis pneumonia and progressive multifocal leukencephalopathy [100]. Progressive multifocal leukencephalopathy (PML), is a rare and mostly lethal encephalitis caused by the polyomavirus JC. Both, NHL and chemotherapy are risk factors for developing PML. Therefore, the additive risk for this unusual infectious complication by adding rituximab to the treatment of NHL patients remains to be quantified. Incidence of PML is likely to be exceedingly low with rates of 0.1–4.3 for every 1,000 patient years for NHL patients exposed to rituximab [13,93]. Rituximab associated PML cases and unusual viral infections in patients with lymphoproliferative disorders are summarized in Table 5.

The pivotal pediatric study already mentioned above analysed infectious events of 2875 children. Critical complications of opportunistic infections within one year after rituximab exposure were low. Three patients had a discharge diagnosis code for *P. joroveci* pneumonia, one patient with hepatitis B and one with progressive multifocal leukoencephalopathy.

## 3. Summary, Conclusions and Perspectives

Rituximab might offer opportunities to optimize available pediatric chemotherapy regimens in sense of reducing toxicity and prevention of relapse in pediatric B-cell lymphoma. Given the paucity of data regarding rituximab use in children, the reviewed data provide an idea of the expected kinetic of immunreconstitution and the risk of infectious complications in pediatric and adolescent patients. Overall, the available data hint at a similar kinetic of immunreconstitution in pediatric and adult patients with the start of recovery after six months and normalization one year after therapy. Whether this transient depletion of B-cells is associated with an increased risk for infections remains open but the available data indicate only a minimal additional risk in adults. There is evidence that the risk of infections significantly depends on the additional chemotherapeutic treatment, the underlying disease and the status of the immune system.

Controlled systematic clinical trials focused on children and adolescents are urgently indicated to prospectively evaluate the kinetic of immune reconstitution and its clinical relevance taking into account the indication of rituximab treatment and the maturation status of the immune system. One additional aim must be to identify the characteristics of the individual patients who do not recover after rituximab treatment and to prohibit rituximab treatment in these children whenever possible.
